# Myelofibrosis and allogeneic transplantation: critical points and challenges

**DOI:** 10.3389/fonc.2024.1396435

**Published:** 2024-06-20

**Authors:** Paola Ranalli, Annalisa Natale, Francesco Guardalupi, Stella Santarone, Chiara Cantò, Gaetano La Barba, Mauro Di Ianni

**Affiliations:** ^1^ Hematology Unit, Pescara Hospital, Pescara, Italy; ^2^ Department of Medicine and Aging Sciences, University of Chieti-Pescara, Chieti, Italy

**Keywords:** myelofibrosis, bone marrow transplantation, JAK inhibitors, scoring algorithm, splenomegaly

## Abstract

New available drugs allow better control of systemic symptoms associated with myelofibrosis (MF) and splenomegaly but they do not modify the natural history of progressive and poor prognosis disease. Thus, hematopoietic stem cell transplantation (HSCT) is still considered the only available curative treatment for patients with MF. Despite the increasing number of procedures worldwide in recent years, HSCT for MF patients remains challenging. An increasingly complex network of the patient, disease, and transplant-related factors should be considered to understand the need for and the benefits of the procedure. Unfortunately, prospective trials are often lacking in this setting, making an evidence-based decision process particularly arduous. In the present review, we will analyze the main controversial points of allogeneic transplantation in MF, that is, the development of more sophisticated models for the identification of eligible patients; the need for tools offering a more precise definition of expected outcomes combining comorbidity assessment and factors related to the procedure; the decision-making process about the best transplantation time; the evaluation of the most appropriate platform for curative treatment; the impact of splenomegaly; and splenectomy on outcomes.

## 1 Introduction

Myelofibrosis (MF) includes primary myelofibrosis (PMF) and secondary myelofibrosis (SMF). The latter includes post-essential thrombocythemia (PET) and post-polycythemia vera (PPV) myelofibrosis. PET and PPV are associated with inferior overall survival (OS) rates compared to PMF, often due to its higher risk of leukemic transformation (LT). According to WHO and International Consensus Classification of Myeloid Meoplasms and Acute Leukemias (ICC’s) current diagnostic criteria, PMF may present two different clinical pictures: prefibrotic or early myelofibrosis (pre-PMF) and overt myelofibrosis, differing from each other essentially in the bone marrow grade of fibrosis (1–3).

MF treatment options are still limited. The treatment of low-risk MF is generally related to symptom severity. The treatment of high-risk diseases includes JAK inhibitors (JAKi) and allogeneic hematopoietic stem cell transplantation (HSCT). HSCT remains the only chance of a definitive cure for patients with both primary and secondary myelofibrosis (4). After a failure of conventional treatments, in MF patients ineligible for HSCT, enrollment in clinical trials represents an alternative option, when available.

Guidelines recommend upfront allogeneic bone marrow transplantation in patients with high-risk disease, following data from a retrospective study showing patients with intermediate-2 or high-risk score in the Dynamic International Prognostic Scoring System (DIPSS) who benefited the most from transplant than conventional therapy only (5–8).

Thus, an accurate assessment of MF-related risk should be provided by clinicians to promptly identify patients with < 5 years of expected survival, potentially candidates for hematopoietic cell transplantation. Information about transplant-related morbidity and mortality, the expected post-HSCT outcome, as well as MF-related risk, should be adequately shared with patients and their families. Such an integrated evaluation may allow proper counseling about global post-transplant prognosis. Finally, the decision has to be always taken on an individual basis (9).

Moreover, novel strategies for patient and donor selection, conditioning regimens, and post-transplant care in the last years allowed the reduction of disease relapse incidence, 5-year non-relapse mortality and survival in related and unrelated donor transplants in patients with myeloproliferative neoplasms (10), thus, justifying allo-HSCT as a curative option in younger patients of all risk categories and not just in high-risk diseases (11, 12) or in carriers of high-risk non-driver mutations (EZH2, ASXL1, IDH1/2, and SRSF2), predictive of inferior OS and disease-free survival (DFS) (13).

HSCT for MF patients remains challenging, particularly in older age patients or in those with cytopenias, splenomegaly, and severe bone marrow fibrosis (14).

Certainly, the global number of HSCTs in MF rose recently, signifying the increased interest in the only available curative treatment for the disease.

In the present review, we will analyze the main controversial points concerning allogeneic bone marrow transplantation in myelofibrosis.

## 2 Age at transplant

Older patients are often carriers of metabolic or systemic comorbidities making them more vulnerable to toxicity associated with treatment (10, 15). In studies with a long follow-up, the recipient’s age was shown to have an impact on overall survival (OS) and on the risk of treatment failure (16). In a single-center retrospective study on patients with MF who underwent a reduced intensity conditioned (RIC) HSCT, older male patients reported an increased incidence of poor graft function (P = 0.05). This phenomenon was not associated with an increased risk of relapse/progression and did not impact OS (17).

Accordingly, age has been considered the most important factor adversely impacting transplant outcomes in myeloproliferative neoplasms (MPN) (17, 18) and clinicians have been traditionally reluctant to offer the procedure to older patients.

The age of 70 represented the upper limit established in European Society for Blood and Marrow Transplantation (EBMT)/Europena Leukemia Net (ELN) recommendations to proceed with allogeneic transplantation in subjects with intermediate-2 or high-risk disease (19).

Recently, encouraging results were shown in studies involving older patients (>70) undergoing HSCT for MF (20). Engraftment, rates of graft-vs. -host disease (GvHD), progression-free survival (PFS), and OS comparable to those reported in younger patients were shown in selected patients with primary or secondary myelofibrosis aged 60 to 78 years old who received HSCT from Human Leukocyte Antigen (HLA)-identical siblings or unrelated donors (21). Thus, age should not represent an absolute contraindication to allo-HSCT, in case of absent or well-controlled comorbidities (6).

The use of RIC regimens allow more favorable survival in older patients aged > 65 years old with no or minimal comorbidities (22). EBMT/ELN recommendations suggest considering the possibility of HSCT case by case, taking into account patients’ and disease variables and also the recipient’s preferences (6).

## 3 Selection of patients

### 3.1 Stratification of the risk associated with MF

Traditional prognostic models include the International Prognostic Scoring System or IPSS (only applicable to newly diagnosed patients) (23); the dynamic IPSS or DIPSS (24) (applicable at any time point after diagnosis); and the DIPSS plus (25). They all include clinical parameters only (age, anemia, leukocytosis, circulating blasts, and constitutional symptoms), each independently predicting inferior survival. DIPSS plus also includes thrombocytopenia (platelets <100 × 10^9^/L), unfavorable karyotyping (traditionally established), and the need for transfusion support (26).

In the pre-ruxolitinib era, only high-risk patients seemed to gain the greatest survival advantage from transplantation. A retrospective multicenter study including 438 patients with primary or PET and PPV myelofibrosis aged less than 65 years clearly showed a significantly lower risk of death after HSCT in comparison with subjects treated with conventional therapies only in case of DIPSS intermediate-2 and high-risk patients (respectively, p: 0.005 and 0.0007 vs. conventional therapies) (5).

Scoring systems including more prognostic parameters, mainly molecular data, were later introduced (**Table 1**). Among driver mutations, it has become clear that CALR, particularly type I mutation, is associated with a more indolent course, thus its protective function against progressive disease has been recognized and with better post-transplant outcome [higher 4-year OS and lower 4-year non-relapse mortality (NRM) after allo-HSCT] (27–29). On the opposite, the worst prognosis is associated with the JAK2/CALR/MPL triple-negative profile (30) (31).

**Table 1:** Variables included in prognostic scores applied in myelofibrosis and identification of patients with poor OS.

	Dynamic International Prognostic Scoring System (DIPSS)	Dynamic International Prognostic Scoring System (DIPSS-Plus)	Genetically Inspired Prognostic Scoring System (GIPSS)	Mutation-Enhanced International Prognostic Scoring System Plus Karyotype (MIPSS70 + 2.0)
**Age**	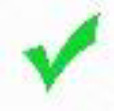	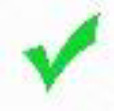	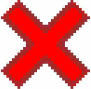	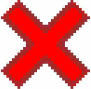
**Constitutional symptoms**	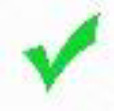	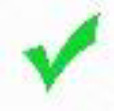	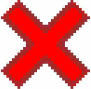	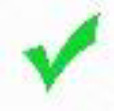
**Blood count values**	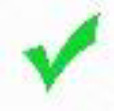	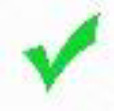	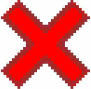	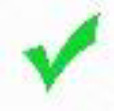
**Blasts in peripheral blood**	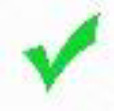	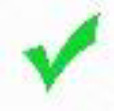	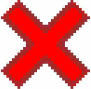	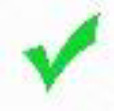
**Karyotype**	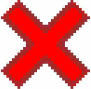	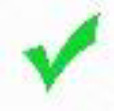	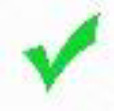	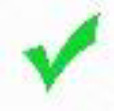
**Driver mutations**	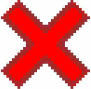	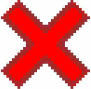	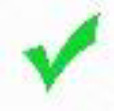	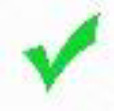
**Non-driver mutations**	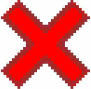	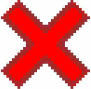	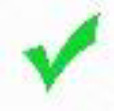	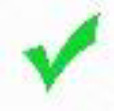
Expected overall survival for each class of risk *	**• LR→ not reached** **• Int-1→14.2 years** **• Int-2→4 years** **• HR→1.5 years**	**• LR→185 months** **• Int-1→78 months** **• Int-2→35 months** **• HR →16 months**	**• LR →26.4 years** **• Int-1→10.3 years** **• Int-2→ 4.6 years** **• HR→2.6 years**	**• VLR→ not reached** **• LR→7 years** **• HR →3.5 years** **• VHR →1.8 years**

*Expected overall survival for each class of risk according to a single stratification risk system: for DIPSS, DIPSS plus, and GIPSS: LR, low risk; Int-1, intermediate 1; Int-2, intermediate 2; HR, high risk. For MIPSS70 + 2.0: VLR, very low risk; LR, low risk; HR, high risk; and VHR, very high risk.

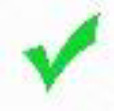
, variable included; 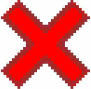, variable not included.

Among non-driver mutations, ASXL1, EZH2, IDH1/2, and SRSF2 were identified as mutations associated with a poor prognosis, defining the group of “high molecular risk (HMR) mutations,” linked with poorer prognosis (31–33). The Mutation Enhanced International Prognostic Score Systems (MIPSS) were designed for HSCT decision-making in patients aged ≤70 years.

MIPSS70 identifies as significant risk factors for OS both clinical factors (anemia, leukocytosis, thrombocytopenia, constitutional symptoms, and circulating blasts ≥2%) and nonclinical conditions, not included in the previous traditional models, such as bone marrow fibrosis grade ≥2, absence of CALR type-1 mutation, presence of high-molecular risk mutation (ASXL1, EZH2, SRSF2, and IDH1/2), and the number of two or more high-molecular risk mutations.

The MIPSS70-plus is enriched with cytogenetic information (34), adding a more refined definition of cytogenetic risk, thus providing three risk categories. MIPSS70-plus version 2.0 represents a more complex system including more non-driver mutations (U2AF1) and also considering the number of high-risk mutations and a three-tiered cytogenetic risk classification as independent prognostic factors (34–37). New prognostic thresholds considering severity and sex-adjusted values for hemoglobin levels were also integrated (38).

Genetically inspired prognostic scoring system for primary myelofibrosis (GIPSS) focuses only on genetic and molecular factors, particularly on a limited number of non-driver lesions, without considering clinical parameters at all (39).

Patients may be identified for the HSCT path also according to the best response obtained to pharmacological therapy used as “bridge to transplant,” usually JAKi. Lower rates of responses to ruxolitinib have been shown in cytopenic vs. non-cytopenic MF, making cytopenic patients more often considered for earlier HSCT (40).

Moreover, in a recent retrospective study, a positive correlation has been found between peripheral blood CD34 cells and spleen length in both PMF and SMF, thus identifying a possible tool facilitating the assessment of spleen response more objectively than deep palpation of the abdomen (41).

Discrepancies emerged from the application of standard prognostic scores in patients with secondary MF (42). MYelofibrosis SECondary to PV and ET prognostic Model (MYSEC-PM) was validated as the only specific prognostic tool suitable for patients with MF secondary to PV and ET including both clinical and molecular data (43).

Disagreement between modern prognostic scores and traditional scores based on only clinical parameters have been observed (34, 44). GIPSS and clinical-only scores may differ quite frequently as they do not share any variable. That is why at the same risk class, an inferior OS and a worse leukemia-free survival (LFS) were shown in genomically vs. clinically established higher-risk patients (p = 0.08 and p = 0.04, respectively) (44).

To ensure the most complete evaluation of disease-associated risk, a simultaneous assessment of as many scores as possible could be facilitated by a PMF-specific calculator (45). However, recent EBMT/ELN recommendations indicate allogeneic HSCT based exclusively on DIPSS, MIPSS70, and MIPSS70 plus scores (6).

Many other disease-specific factors have an impact on the outcome as increased circulating CD34+ cells, increased bone marrow or circulating blasts (46, 47), TP53, CBL, N/KRAS mutations (48), triple negativity (31), cytopenic PMF (49). The latter is associated with both higher rates of leukemic transformation and worse survival, and generally, it pairs up with JAK2 V617F allele burden, less prominent splenomegaly, greater genomic complexity and increased risk for infections and bleeding (50).

Splenomegaly itself is not included among the relevant parameters of the prognostic scores used for myelofibrosis despite the fact that larger baseline spleen volume correlates with an increased risk of death in the COntrolled MyeloFibrosis study with ORal jak inhibitor Treatment (COMFORT) studies (51).

Studying a large cohort of patients with MPN (n = 2,035), not necessarily with MF, Grinfeld et al. identified distinct subgroups of MF patients with distinct clinical, cytogenetic, and mutational features, thus developing a personalized MPN risk calculator predicting survival and leukemic transformation, and demonstrating for the first time, the detrimental effect of mutated TP53 on survival in MF patients (52).

### 3.2 Prediction of post-HSCT outcomes

The role of current prognostic systems in predicting outcomes after HSCT is still uncertain (12, 53–55). DIPSS and DIPSS plus have been shown as predictive tools also for survival following HSCT despite not including the evaluation of transplant-specific variables (5, 53, 56). In SMF, MYSEC was predictive also for survival after allogeneic HSCT, as shown in a recent study (57).

A recent retrospective study published this year by Polverelli et al. on behalf of the Chronic Malignancies Working Party of EBMT confirmed the relevant and negative impact of comorbidities on HSCT outcomes for patients with MF, underlining the need to integrate such an information in the selection process (58).

The hematopoietic cell transplantation-specific comorbidity index, better known as Sorror index provides a reliable scoring of pretransplant comorbidities to more precisely define both non-relapse mortality (NRM) and survival (OS), showing a better prediction power than the Charlson Comorbidity Index (CCI). It is usually applied to all hematological diagnoses, MF included, despite having patients diagnosed with MF who were not included in the validation cohort (59).

Undoubtedly, patient- and transplant-specific risk factors like the intensity of the conditioning regimen, recipient age, cytomegalovirus serostatus, performance status or HLA matching of the donor, influence the patient’s post-HSCT outcome (19, 60).

“Myelofibrosis transplant scoring system” or MTSS, a four-level clinical-molecular score including clinical data, donor type, and mutation status for ASXL1/CALR/MPL, has been validated as a specific prognostic tool for an objective evaluation of the risk/benefit ratio of HSCT in the counseling phase, before transplantation (61).

MTSS identified independent risk factors for poor survival after transplant (pretransplantation thrombocytopenia, leukocytosis, older age, poor performance according to Karnofsky performance status, a non-CALR/MPL driver mutation genotype, ASXL1-mutation and transplantation from an HLA-mismatched unrelated donor). It should be noted that it does not include significant risk factors (from existing scoring systems) like anemia and transfusion dependency, constitutional symptoms, cytogenetic risk stratification, or the presence of two or more HMR mutations (61).

Unfortunately, the MTSS scoring system did not maintain its predictive role in other series of cases (62). Another limitation on the use of MTSS is the lack of information about comorbidities. Therefore, the application of MTSS does not disregard the need for a comorbidity index evaluation as well.

Outside the MTSS score, other variables such as spleen size, transfusion history, donor type (11), JAKV617F status, age, and constitutional symptoms are predictive of 5-year OS (55).

Recently, a detrimental effect on transplant outcome was shown in carriers of TP53 mutations of a large multicenter cohort. In particular, higher mortality was demonstrated as a consequence of higher rates of early leukemic transformation, almost a case of “*multi-hit constellation”* (63).

### 3.3 Final decision about HSCT

In conclusion, there is experts’ consensus on the eligibility to transplant for intermediate-2/high-risk DIPSS patients, high-risk MIPSS70 or MIPSS70-plus, high-risk or intermediate-2 MYSEC-PM who, at the same time, present a low to intermediate-risk profile according to MTSS.

Allogeneic HSCT should also be offered to DIPSS intermediate 1 risk patients and to MIPSS70 or MIPSS intermediate patients who present a low-risk profile with MTSS, taking into great consideration patients’ preferences, response to treatment, and other issues such as availability of clinical trial or additive data (6).

Traditionally, variables to consider in non-high-risk patients with MF, suggesting eligibility for transplant are represented by (1) transfusion-dependent anemia, (2) a percentage of blasts in peripheral blood > 2%, (3) adverse cytogenetics, and (4) high-risk mutations (64).

## 4 Splenomegaly and splenectomy

Splenomegaly is a hallmark of both primary and PET and PPV myelofibrosis, as it represents the malignant clone expanding outside the bone marrow. The real impact of spleen size and eventual splenectomy on HSCT outcomes in myelofibrosis is still debated.

Several studies have shown that splenomegaly can adversely impact transplant outcomes, as it may promote the sequestration of hematopoietic progenitors (65, 66).

In a retrospective study involving a limited number of patients with myelofibrosis, the authors considered massive splenomegaly as one of the variables adversely affecting the outcome of HSCT. This variable was included in a scoring tool used for decision-making, alongside other variables such as a transfusion history of > 20 red blood cell units before transplantation and the type of alternative donor (11).

The effect of a huge spleen on OS and relapse after allo-HSCT is not completely clear, as conflicting data emerged from other works (60, 67, 68).

Furthermore, splenomegaly was associated with a higher risk of relapse after transplantation in recent studies (69, 70).

Potentially, splenectomy before HSCT could be useful for disease debulking and also to favor a faster hematopoietic recovery (66, 71) but some other data, in contrast, did not confirm it (12, 72).

A retrospective EBMT study on 1,000 cases of MF splenectomized in comparison with non-splenectomized patients, did not report a different OS (P = 0.274) rate, but the results seemed associated with a lower rate of NRM (P = 0.018) and increased risk of relapse (P = 0.042). However, in a subanalysis considering splenectomy in different subgroups of patients, an improved outcome was reported with splenectomy in subjects with a palpable spleen length ≥ 15 cm (better OS, significant reduction in NRM, not significantly increased relapse risk, P <.001, P <.001, and P = .147 for each phenomenon) (73).

How splenectomy affects the risk of disease relapse and survival after HSCT is still unclear, thus, making it mandatory for future more prospective randomized trials.

Moreover, data available from retrospective studies on GvHD in previously splenectomized patients are quite conflicting (65, 67, 74).

The course of splenectomy can be complicated by thrombosis, bleeding, infections in up to 30% of patients, disease transformation, and death (peri-operative mortality is in the range of 5%–10%) (75). All complications eventually preclude or simply delay allo-HSCT (72, 76–78).

A multicenter retrospective study on 530 patients with a diagnosis of myelofibrosis from the French bone marrow transplantation registry (RFGM) who underwent splenectomy in the period 2008–2017 showed reassuring results, as pretransplant splenectomy did not preclude allo-HSCT; in particular, splenectomized patients had a higher rate of transplantation in the first 4 months after splenectomy [HR (95% CI) = 7.2 (5.1–10.3)] but not after this time point (79).

As spleen size in patients with MF sensitively benefit from JAKi (80–82), the need for splenectomy has to be discussed rarely nowadays.

Despite not being routinely performed or recommended, splenectomy remains useful in patients who did not benefit from therapy with JAKi, with residual massive splenomegaly.

Splenic irradiation represents a further alternative to splenectomy to reduce spleen size and alleviate splenic discomfort, although the results of such therapy are generally short-lasting and associated with the risk of severe cytopenias, eventually difficult to manage (83).In contrast, the results of a recent retrospective study on HSCT for MF preceded by splenic irradiation are encouraging, showing a reduced relapse after HSCT, without association between total irradiation dose and efficacy (84).

Therefore, it may be considered for patients not eligible for surgery or who were no longer responsive to JAKi (85, 86).

The impact of splenic radiotherapy in leukemic transformation (LT) is still unclear; conversely, similar engraftment rates and GvHD incidence have been described in patients who underwent splenic irradiation or not (74).

## 5 JAKi and timing

The option of upfront HSCT is still recommended for patients stratified as intermediate 2 and high-risk DIPSS, with an expected survival of fewer than 5 years (6), as it provides the best gain in life expectancy. This indication was reinforced by a decision analysis recently published (87).

In the case of a patient with intermediate-risk disease, the decision about HSCT requires a more tailored approach, and generally, the procedure can be delayed; usually, in this setting, more prognostic factors, even outside traditional scores, have to be considered, identifying those patients less likely to have lasting response from non-transplant therapy (6, 88).

The best timing of HSCT has become a more controversial point in the era of JAKi as these drugs produce a better action on spleen size, constitutional symptoms, and also on survival outcomes.

Studies showed better response in patients treated earlier during the disease course with both HSCT (56) and JAKi, thus, decision-making about transplant becomes even more complex. It should be underlined that JAKi are not curative (89, 90), and they do not prevent the progression to blast phase or leukemic transformation, the main determinant of death in MF (23, 91, 92).

Furthermore, despite the success with ruxolitinib in the frontline setting, discontinuation of JAKi therapy may occur because of intolerance or refractoriness, events associated with a poor OS according to retrospective studies (93, 94).

Comparative studies testing the results of upfront HSCT approaches with non-transplant therapies of the JAKi era are still lacking, thus leading to a wide variability of conducts on the use of HSCT in MF. In 2024, upfront JAKi therapy was compared with upfront HSCT strategy in MF patients not older than 70 years old in a large, multicenter and retrospective study; in patients treated with upfront HSCT, an earlier mortality was observed and in general, they do not report significative benefit (95).

Thus, one can imagine that in the “JAKi era,” HSCT is limited to cases of cytopenic myelofibrosis, not manageable with cytoreductive or JAKi therapy; could be delayed until response to JAKi is lost, and that delaying time could become even longer as more than one JAKi has become available (21). In fact, according to some recent data, fedratinib or other JAKi may improve upon the poor prognosis associated with ruxolitinib discontinuation (96, 97).

Advanced-stage disease, increasing age, or leukemic transformation, often associated with the emergence of acquired unfavorable mutations, could represent the dramatic consequences of delaying the HSCT procedure, as impactful disease-modifying therapy other than HSCT still does not exist.

For this reason, many authors underline that patients whose therapeutic goal is cure should still undergo HSCT even if responding to JAKi (98).

The possibility to rapidly obtain a spleen response with JAKi represents an attractive option for clinicians looking for a “bridge to transplant strategy,” thus eventually making engraftment time more rapid (99, 100). The use of JAKi as pre-HSCT strategy is increasing and offers encouraging results. With this approach, eligible patients should undergo HSCT at the time of the best response to JAKi (100).

In non-randomized and retrospective studies, the treatment of patients with ruxolitinib in the phase preceding HSCT is well-tolerated and associated with better post-transplant outcomes and survival (101, 102). The prospective phase-2 trial JAK ALLO study showed that a short course of ruxolitinib administered before HSCT and stopped progressively or abruptly before the conditioning regimen is safe and associated with a high probability of HSCT for those with a donor and no increased risk of disease progression (103).

The initiation of ruxolitinib is recommended ≥2 months before HSCT, careful weaning 5–7 days before conditioning, and complete withdrawal on the day before conditioning according to the European guidelines for primary MF (19). Adverse events happened in patients who stopped JAK inhibitor ≥ 6 days before conditioning therapy (104), while they were infrequent in those treated with JAK inhibitor until HSCT conditioning therapy was started (105).

Future studies will clarify the hypothesis that JAKi treatment in candidates for HSCT may reduce the incidence of poor graft function (17).

It should be noted that in MF patients pre-treated with ruxolitinib for 6 months before HSCT, different outcomes were shown according to the type of donor. In particular, poorer mortality and GvHD outcomes were associated with patients receiving HSCT from an unrelated donor compared to those with a matched sibling donor in JAK ALLO phase-2 trial. These results could be explained by many factors such as advanced disease, loss of response to ruxolitinb at the time of HSCT or insufficient period of treatment, thus not showing a direct impact of ruxolitinib on post-HSCT outcomes (103).

In a multicenter German study reporting the experience of ruxolitinib pretreatment in 159 MF patients who underwent RIC HSCT between 2000 and 2015 from different types of donors, ruxolitinib did not negatively impact HSCT outcomes, as similar outcomes were shown in non-ruxolitinib pre-treated patients. Similar OS, DFS, and GvHD were reported among ruxolitinib responders and those who failed to respond or were no longer responsive to JAKi (106).

Following JAKi failure (93, 107), HSCT should be considered in any patient (108), according to little data from retrospective studies showing improved survival with HSCT in this setting (109).

The treatment landscape has become more intricate with the availability of fedratinib and novel combination strategies involving ruxolitinib within clinical trials. Nevertheless, HSCT remains a viable option for eligible candidates. Despite the efficacy of fedratinib on splenomegaly, there is still lack of information on the use of this agent or other novel agents, as an alternative to ruxolitinib, before HSCT (6).

Thus, response to ruxolitinib should be systematically assessed 6 months after initiating therapy (6), as recently recommended by EBMT/ELN.

The model, named Response to Ruxolitinib After 6 Months (RR6), was validated as a prognostic model allowing the identification of MF patients who have already been treated with ruxolitinib for 6 months and in need of second-line treatment strategies, HSCT included. The predictive role of such a prognostic tool, evaluating three variables (drug dose, spleen response, and transfusion requirement) and thus stratifying the risk into three categories (low, intermediate, and high) overcomes conventional risk stratification in MF treated with ruxolitinib (110). According to the RR6 model, high-risk patients need a prompt evaluation for HSCT (6).

## 6 Identification of stem cells donors

Donor type is an important predictor of outcome for MF transplanted patients, with HLA-matched sibling donors (MSD) being preferred over matched unrelated donors (MUD) and mismatched unrelated donors (mMUD). Gupta et al. reported HSCT outcomes of 233 MF patients for CIMBTR. In multivariate analysis, donor type was the sole independent factor associated with survival (5-year OS was 56%, 48%, and 34% for MSD, MUD, and mMUD, respectively) (111).

Alternative donor options in MF expand the donor pool in patients who do not have a suitable sibling or unrelated donor. Unrelated cord blood units are rarely used in MF patients, with graft failure remaining a major concern. A retrospective study from the EBMT registry evaluated 35 patients who received cord blood HSCT reporting 2 years of OS and EFS rates being 44% and 30%, respectively (112).

The haploidentical setting is still under investigation with improving results over time. Bregante et al. evaluated the outcome of 95 patients with myelofibrosis who were allografted between 2001 and 2014. The 3-year HSCT-related mortality (TRM), relapse rate, and overall survival were 16% vs. 32%, 16% vs. 40%, and 70% vs. 39%, respectively, in the 2011 to 2014 period versus the 2000 to 2010 period. Improved survival was most pronounced in alternative donors (69% vs. 21%), compared with MSD (72% vs. 45%) (113).

Kunte et al. reported the results from a multicenter retrospective study of 69 patients who underwent haploidentical HSCT with post-procedural cyclophosphamide (PTCy) with 3-year OS being 72%, 3-year relapse-free survival of 44%, and non-relapse mortality of 23% (69).

## 7 Primary graft failure and poor graft function

Primary graft failure and poor graft function are two difficult challenges after HSCT.

Primary graft failure is defined according to the EBMT criteria by an ANC < 0.5 × 10^9^/L by day +28 following stem cell infusion, Hb <8.0 g/L, and platelets <20 × 10^9^ (114).

In a retrospective EBMT study involving 2,916 MF patients who underwent allo-HSCT from an HLA-identical sibling or unrelated donor between 2000 and 2016, the 5-year survival rate in patients who developed graft failure was 14% (115).

Recognized risk factors for transplanted patients are related to donor, conditioning, cell dose, and HLA sensitization if the recipient is heavily transfused (116).

No consensus is available about therapeutic options for patients with graft failure, second allo-HSCT using either the same or alternative stem cell donor is warranted (116).

Recently, a retrospective study from the Francophone Society of Bone Marrow Transplantation and Cellular Therapy demonstrated the rescuable potential of salvage haplo-HSCT with PTCy for graft failure. The median time to neutrophil engraftment was 18 days and the cumulative incidence of neutrophil engraftment at day 30 was 79%. One-year overall survival (OS) was 56% and HSCT complications accounted for 80% of causes of death, with multiple organ failure as the leading cause (117).

According to the EBMT criteria, poor graft function (PGF) is defined by the presence of bi- or tri-lineage cytopenia lasting for more than 2 weeks, after day +28 in the presence of donor chimerism >5% (114).

Moreover, PGF is also defined by the presence of mild/moderate cytopenias in at least two hematopoietic lines (ANC < 1.5 × 10^9^/L, platelet count < 30 × 10^9^/L, Hb < 8.5 g/dL) lasting for more than 2 consecutive weeks following engraftment beyond day +14. This definition was recently introduced by an expert panel of the EBMT Chronic Malignancies Working Party, because it is easier to apply in clinical practice than the former one (116).

In a cohort of 100 patients with primary MF or post-ET/PV MF who received a reduced-intensity HSCT, the cumulative incidence of poor graft function was 17% and all cases occurred before day 100 after HSCT at a median of 49 days (range: 24–99 days). In univariate analysis, recipients of older age and splenomegaly at day 30 after HSCT showed an increased cumulative incidence of poor graft function (17).

An expert panel from the EBMT/ELN International Working Group recommends the use of growth factors for anemia (erythropoietin) or neutropenia (granulocyte colony-stimulating factor), whereas data on the use of thrombopoietin analogs in patients with myelofibrosis who underwent allogeneic HSCT are scarce. The most definitive treatment for poor graft function is a CD34+ stem-cell boost from the original donor, either fresh or cryopreserved, without further conditioning in patients without active GvHD (6).

In the Hamburg cohort, CD34+ selected stem cell boost infusion in patients with PGF achieved similar outcomes at 3 years when compared to patients who did not have PGF (17).

Management of persistent splenomegaly in patients with PGF after HSCT is challenging. Splenectomy was reported to be an option in selected patients (17) but it is not without risks. JAK2 inhibitors have not been tested for the indication of post-procedural poor graft function. In majority of the patients, tri-lineage hematologic recovery can be achieved, but will require several months.

## 8 Relapse after HSCT

Unfortunately, 10%–30% of transplanted patients experience MF relapse after a median of 7 months after HSCT with a median overall survival from the time of relapse of 2 years (116, 118).

Ataganduz et al. also described a late relapse in 14% of patients later than 5 years after HSCT at a median of 7.1 years (119) (**Table 2**).

**Table 2:** Transplant outcomes in MF patients.

Type of study/Reference	Patients/Follow-up	Outcomes	TRM/NRM
Retrospective multicenter Maze D et al., BMT 2024 (95)	302 patients: 89 upfront HSCT vs. 213 JAKi Median follow-up: 49 months	OS@ 36 months: prior JAKi 69%, upfront HSCT 42%	TRM @ 12 months: 27% HSCT group vs. 3% JAKi group
Retrospective multicenter Hernandez-Boluda et al., BMT 2024 (120)	346 CALR-mutated patients Median follow-up: 40 months	OS @ 1, 3, and 5 years: 81%, 71%, and 63%	TRM @ 1, 3, and 5 years: 16%, 22%, and 26%
Retrospective multicenter Kunte et al., Leukemia 2022 (69)	69 patients, haplo donors with PTCy GvHD prophylaxis Median follow-up: 23 months	OS @ 3 years 72%, @ 1 year 74% RFS @ 3 years 44%	TRM @ 1 year 21%, @ 3 years 23%
Retrospective multicenter Hernandez-Boluda et al., American J Hematol 2021 (20)	556 patients aged ≥65 years Median follow-up: 3.4 years	OS @ 5 years 40% Relapse @ 5 years 25%	NRM @ 5 years 37%
Retrospective multicenter Kroger et al., Leukemia 2021 (121)	551 patients: 277 JAKi pre HSCT, 274 no JAKi	EFS @ 2 years: 68.9% for JAKi pre HSCT vs. 53.7% no JAKi	NRM @ 1 year 22%
Retrospective multicenter McLornan et al., BMT 2021 (122)	4142 patients Median follow-up: 48 months	OS @ 3 years 58% Relapse @ 36 months 22%	NRM @ 36 months 23%
Prospective multicenter Robin M et al., BMT 2021 (103)	64 patients Median follow-up: 31 months	OS @ 12 months 68%, @ 24 months 55% DFS @ 12 months 52%, @ 24 months 46%	NRM @ 12 months 42%, @ 24 months 46%
Retrospective multicenter Lwin Y et al., BBMT 2020 (123)	142 patients Median follow-up: 51.8 months	OS @ 1 year 67%, @ 5 years 57%	NRM @ 100 days 16%, @ 1 year 25%

HSCT, hematopoietic stem cell transplantation; OS, overall survival; JAKi, JAK inhibitors; TRM, transplant related mortality; PTCy, post-transplant cyclophosphamide; GvHD, graft versus host disease; RFS, relapse-free survival; NRM, non-relapse mortality; EFS, event-free survival; and DFS, disease-free survival.

The EBMT Chronic Malignancies Working Party defined MF relapses after HSCT as molecular relapse only, cytogenetic relapse only (rarely reported), molecular and cytogenetic relapse only, and morphological/clinical relapse (116).

The expert panel from the EBMT/ELN International Working Group recommends molecular monitoring by sensitive PCR for one of the driver mutations (JAK2, CALR, or MPL) or highly sensitive chimerism for triple-negative MF after HSCT at 1 month and at 3-month intervals thereafter, for up to 1 year and annual testing thereafter (6).

In case of detection of a molecular relapse, early intervention with the aim of reduction of immunosuppressive therapy and use of adoptive immunotherapy with donor lymphocyte infusions (DLI) can achieve molecular remission avoiding progression to overt hematological relapse in responders (116, 124, 125).

Moreover, Gaglemann et al. showed higher rates of complete molecular remission after DLI for molecular relapse comparing hematological relapse (88% and 60%, respectively) (125).

Second HSCT is a valid option to rescue selected fit patients. Nabergoj et al. for the Chronic Malignancies Working Party of EBMT analyzed 216 patients undergoing a second allo-HSCT for either relapse (56%) or graft failure (31%), achieving 42% 3-year overall survival and 39% relapse-free survival (RFS) (126).

Date are insufficient to recommend the use of JAKi after HSCT as maintenance therapy to prevent relapse and in molecular relapse to prevent overt hematological relapse (6, 116). In patients experiencing hematological relapse after HSCT JAKi represent a valid option to reduce constitutional symptoms and/or splenomegaly (127).

Results of transplant outcomes in MF are showed in **Table 2** (20, 69, 95, 103, 120–123). 

## 9 Conclusions

HSCT remains a challenging and controversial procedure in MF; the assessment of the opportunity and modality of HSCT is usually carried out taking into account specific disease variables but also the recipient’s conditions and preferences, case by case.

As the number of HSCT rises rapidly, the best approach to patient and donor selection, splenomegaly management, and timing of HSCT in the era of new drugs need to be clarified. Further studies are required and will test, last but not the least, how to improve HSCT outcomes in this setting. The most appropriate transplant platforms, GvHD prophylaxis, infections management, and thrombosis prophylaxis need to be addressed undoubtedly, as soon as possible.

## Author contributions

PR: Writing – original draft, Writing – review & editing. AN: Writing – original draft, Writing – review & editing. FG: Writing – original draft. SS: Writing – review & editing. CC: Writing – review & editing. GL: Writing – review & editing. MD: Writing – original draft, Writing – review & editing.
